# Imaging heterogeneity in the mitochondrial redox state of premalignant pancreas in the pancreas-specific PTEN-null transgenic mouse model

**DOI:** 10.1186/2050-7771-1-6

**Published:** 2013-01-17

**Authors:** He N Xu, Shoko Nioka, Lin Z Li

**Affiliations:** 1 Department of Radiology, Perelman School of Medicine, University of Pennsylvania, Philadelphia, PA, USA; 2 Britton Chance Laboratory of Redox Imaging, Johnson Research Foundation, Department of Biochemistry and Biophysics, Perelman School of Medicine, University of Pennsylvania, Philadelphia, PA, USA

**Keywords:** Mitochondrial redox state, PTEN-null pancreas, Heterogeneity, NADH, Fp/FAD, Flavoprotein, Imaging, Metabolism, Precancer, Redox scanner

## Abstract

**Background:**

Metabolic alteration is one of the hallmarks of carcinogenesis. We aimed to identify certain metabolic biomarkers for the early detection of pancreatic cancer (PC) using the transgenic PTEN-null mouse model. Pancreas-specific deletion of PTEN in mouse caused progressive premalignant lesions such as highly proliferative ductal metaplasia. We imaged the mitochondrial redox state of the pancreases of the transgenic mice approximately eight months old using the redox scanner, i.e., the nicotinamide adenine dinucleotide/oxidized flavoproteins (NADH/Fp) fluorescence imager at low temperature. Two different approaches, the global averaging of the redox indices without considering tissue heterogeneity along tissue depth and the univariate analysis of multi-section data using tissue depth as a covariate were adopted for the statistical analysis of the multi-section imaging data. The standard deviations of the redox indices and the histogram analysis with Gaussian fit were used to determine the tissue heterogeneity.

**Results:**

All methods show consistently that the PTEN deficient pancreases (Pdx1-Cre;PTEN^lox/lox^) were significantly more heterogeneous in their mitochondrial redox state compared to the controls (PTEN^lox/lox^). Statistical analysis taking into account the variations of the redox state with tissue depth further shows that PTEN deletion significantly shifted the pancreatic tissue to an overall more oxidized state. Oxidization of the PTEN-null group was not seen when the imaging data were analyzed by global averaging without considering the variation of the redox indices along tissue depth, indicating the importance of taking tissue heterogeneity into account for the statistical analysis of the multi-section imaging data.

**Conclusions:**

This study reveals a possible link between the mitochondrial redox state alteration of the pancreas and its malignant transformation and may be further developed for establishing potential metabolic biomarkers for the early diagnosis of pancreatic cancer.

## Background

Pancreatic cancer ranking the fourth leading cause of cancer-related deaths in the United States has the incidence rate approaching the mortality rate [1,2]. The main reason is that at the time of diagnosis 80-85% patients are already present with locally advanced or metastatic disease that prevents curative surgery [3-5]. The etiology of PC is unclear and more than 90% of PC patients acquire the disease sporadically. The early detection of PC is therefore critical to improving the survival rate and reducing the mortality rate. Currently there is no reliable biomarker available for the early diagnosis of PC. Although the most common biomarker for PC, serum carbohydrate antigen 19-9 (CA 19-9) secreted by the exocrine pancreas cells has up to 90% specificity to symptomatic patients, low positive predictive value in asymptomatic patients, false negative results in Lewis negative phenotype, and increased false positivity in the presence of obstructive jaundice limit its usefulness for the early detection of PC [6]. Advances in the technology are of crucial importance to provide the new biomarkers sensitive and specific to PC [7,8]. Since only about 10% PC patients present diagnostic genetic mutations, studies searching for biomarkers for the early detection ought to look beyond these signature mutations.

Cancer metabolism has received renewed research interest in recent years and metabolic alteration has been recognized as a cancer hallmark [9-16]. Metabolic biomarker, such as fluorinated deoxy-glucose (FDG) has been a great aid for the diagnostic purpose of many forms of cancer. However, FDG presents a few limitations when applied to the early detection of pancreatic cancer (PC) [17]. Exploring other cancer biomarkers that are sensitive and specific to PC based on its altered metabolism is obviously worth the effort.

Metabolic changes are reflected in the cellular redox state that is essential for many biological functions including cell growth, differentiation, survival, apoptosis, motility, invasiveness, transcription, and signaling [18]. For instance, alterations in glucose metabolism affect both cytosolic and mitochondrial redox potential [12]. Reprogramming of cellular metabolism by metabolic mutations can also facilitate oncogenesis [19]. Three metabolic enzymes, isocitrate dehydrogenase, succinate dehydrogenase and fumarate hydratase essential in the TCA cycle in the mitochondria acted as an oncogene or tumor suppressors and their mutations were associated with some forms of cancer [19-21]. The reactions catalyzed by these enzymes are driven by the redox potentials, and it is likely that these mutations may also alter the cellular redox state [22].

We previously employed the NADH/Fp fluorescence redox scanning technique [23-27] to probe the possible link between the mitochondrial redox state and tumor progression. NADH and Fp (including flavin adenine dinucleotide FAD) fluorescence are sensitive to the mitochondrial metabolism and their ratios, e.g., Fp/NADH or Fp/(Fp + NADH) are good indicators of the mitochondrial redox state [23,24,28-31]. Our results showed that the mitochondrial redox indices (NADH, Fp, and Fp/(Fp + NADH)) displayed distinct heterogeneities in human melanoma and breast cancer in mouse models and the aggressive tumors have localized regions with more oxidized redox state compared to the indolent ones [18,32-35].

It has also been reported that redox-sensitive autofluorescence imaging could be applied for metabolic characterization of premalignancy. For example, marked changes in the redox state in the dysplastic tissue were observed in one third of human premalignant cervical biopsy samples [36]. Using two-photon technique the redox states of precancerous epithelia of oral cancer were evaluated, showing significant changes for mitochondrial NADH but not for volume-averaged redox ratio (FAD/NADH) [37]. Additionally, researchers imaged the snap-frozen biopsy samples of human cervical tissues using the redox scanner and showed that the dysplastic tissue was significantly more heterogeneous in the mitochondrial redox state in the stromal regions than the normal or inflammatory tissues [38].

However, it is not clear whether redox state change, more specifically to our interest, the mitochondrial redox state alteration, is required for cancer transformation and progression and if so, to what degree. Also, little is known about the interaction between the mitochondrial redox state and the signaling pathways. To investigate whether the mitochondrial redox state alteration is associated with cancer transformation and to identify the potential imaging biomarkers for pancreatic pre-malignancy, we chose to characterize the mitochondrial redox state of a PTEN-null transgenic mouse model [39] by redox scanning. In this model, pancreas-specific PTEN deletion caused pancreatic metaplasia, where the acinar pancreas is progressively replaced with highly proliferative ductal structures and a fraction of the mice develop ductal malignancy. It is known that metaplasia is a harbinger of cancer in many tissues [40-42]. Metasplastic tissue may progress into dysplastic and then malignant neoplastic tissue. Therefore, pancreas-specific PTEN deletion provides an excellent model system to study how the normal tissue is transformed to certain type of PC.

Some of the preliminary results of this study have been reported elsewhere [43]. In this paper, we present the detailed quantitative findings from imaging the mitochondrial redox state in the premalignant pancreas. Our analyses showed that PTEN deletion slightly shifted the mitochondrial redox state to the more oxidized state and caused tissue to become more heterogeneous in the mitochondrial redox state with localized small regions being more oxidized. This work provides initial evidence that the mitochondrial redox imaging indices provide sensitive biomarkers for early detection of PC. Additionally, our work also demonstrates that quantitative analysis accounting for tissue heterogeneity is highly needed to extract relevant rich biological information for tissue imaging whereas volume-average/global average tends to minimize such information.

## Results

### PTEN deletion rendered the pancreases to become more heterogeneous in the mitochondrial redox state

**Figure 1:**
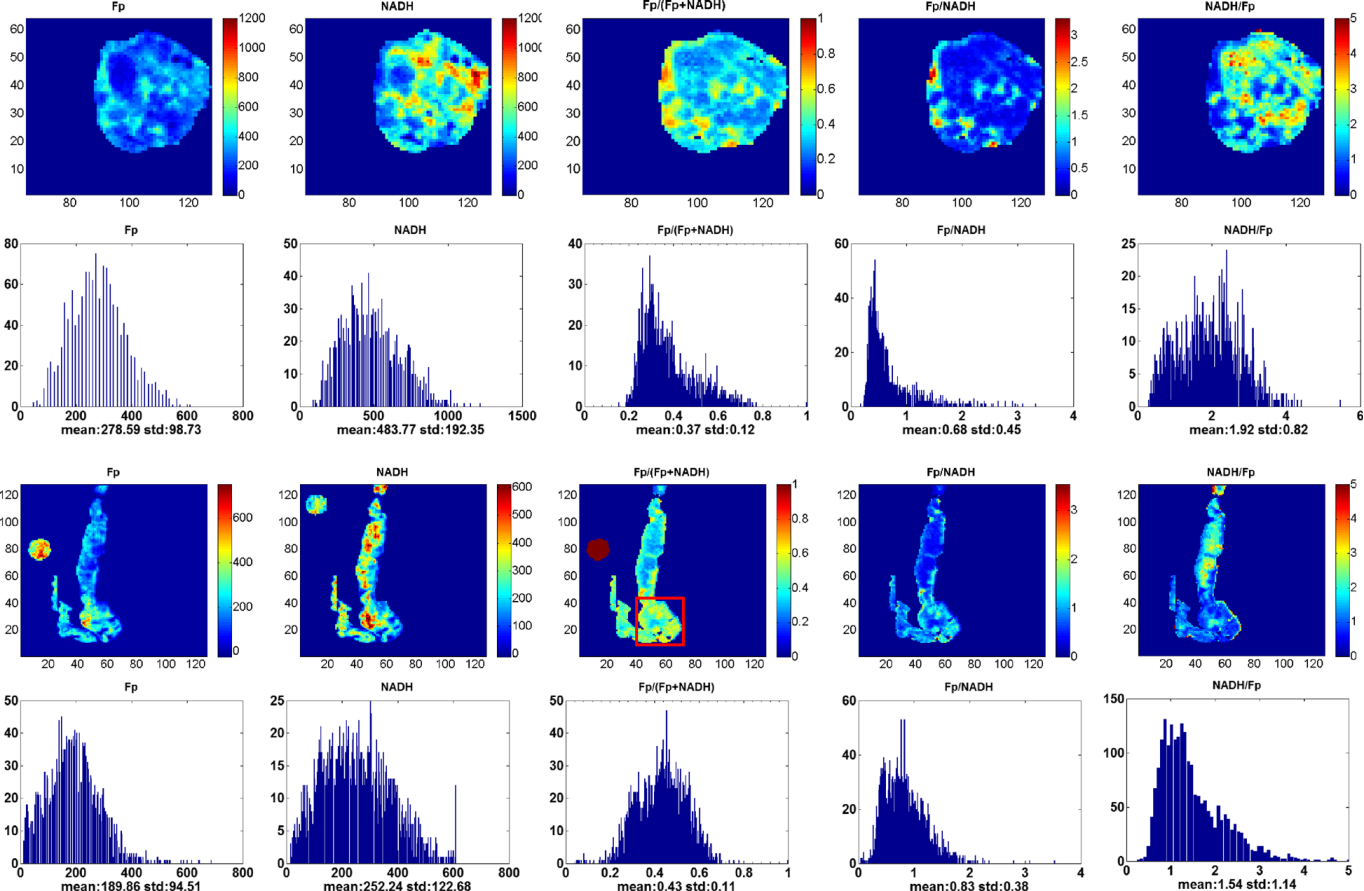
**PTEN-null pancreas (transversal and coronal sections)**. Typical pseudo-color images of the redox indices and their corresponding histograms of a pancreas in the PTEN-null group. The top two rows are the transversal section with a milling depth of 400 μm and the bottom two rows are the coronal section with a milling depth 340 μm. The mean values and SDs are shown on each histogram. From left to right: Fp nominal concentration (μM), NADH nominal concentration (μM), Fp redox ratio, Fp/NADH, and NADH/Fp. The color bars of the Fp and NADH images indicate the nominal concentrations in μM. The color bars of the Fp redox ratio images indicates the ratio range from 0-1. The x-y axes of the pseudo-color images are the dimension of scan matrix, representing the physical location of the tissue relative to the origin of the fiberoptic probe. The x axes of the corresponding histograms represent the Fp or NADH concentration or their ratios. The y axes represent the number of pixels in the tissue section having a specific value of fluorophore concentration or ratio. Image resolution: 100 μm.

**Figure 2:**
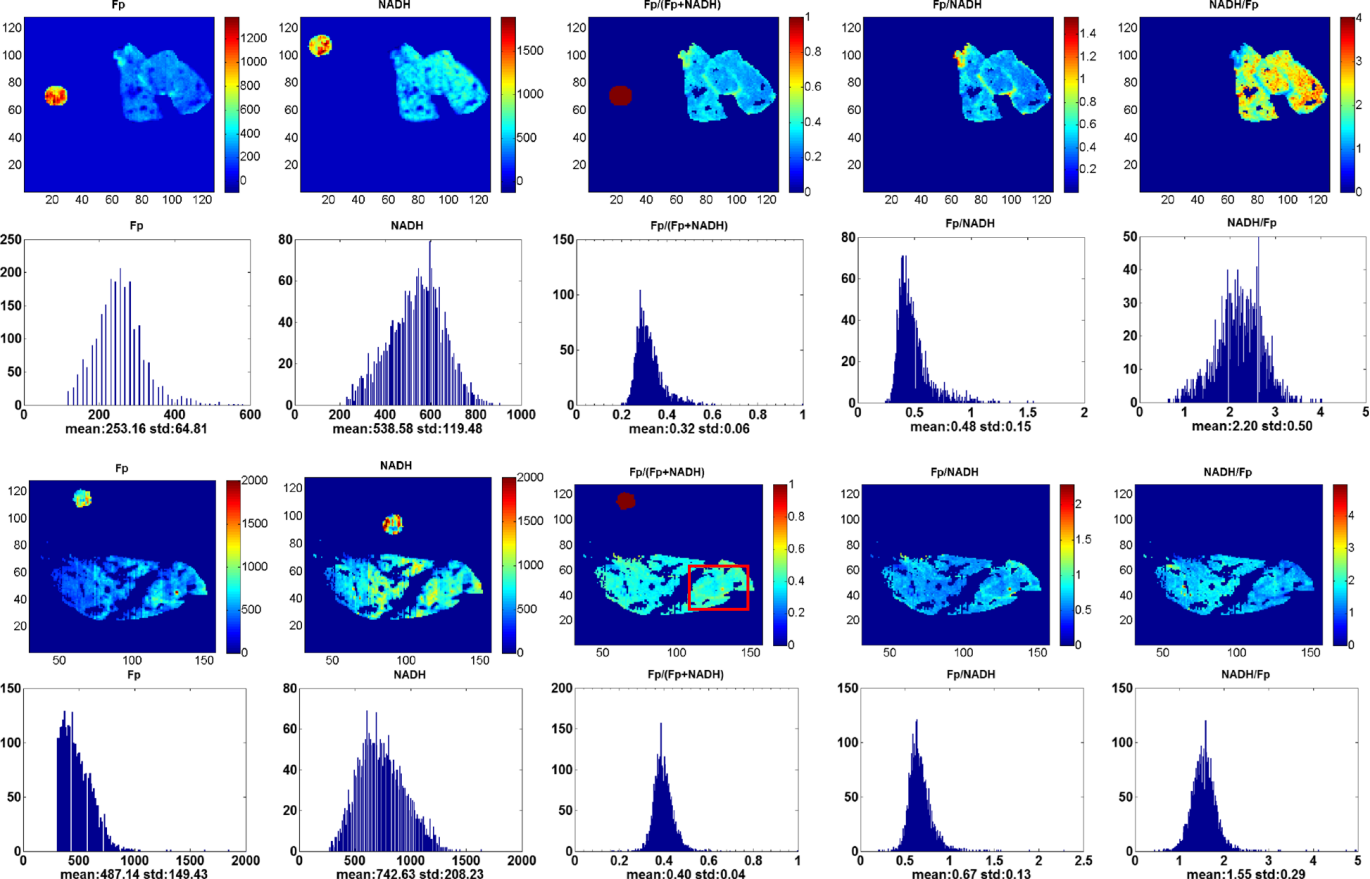
**Control pancreas (transversal and coronal sections).** Typical pseudo-color redox images and their corresponding histograms of a pancreas in the control group. The top two rows are the transversal section with a milling depth 800 μm and the bottom two rows are the coronal section with a milling depth of 800 μm. Image resolution: 100 μm.

**Figure 3:**
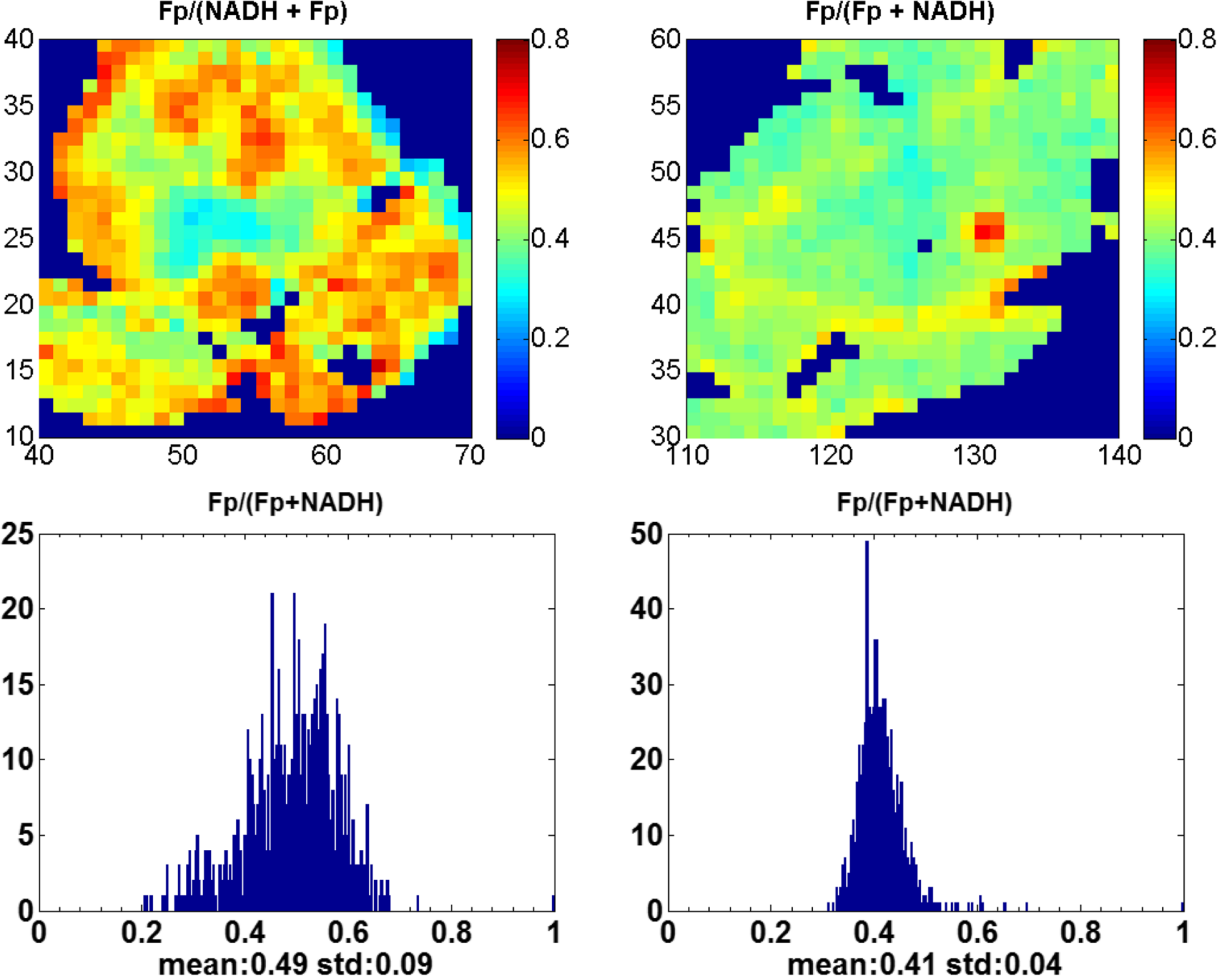
**The magnified areas indicated in the red squares of the Fp redox ratio images in Figures**1**&**2**.** Left-PTEN-null; Right-Ctrl.

We set out to examine the relationship between the premalignant pancreatic tissues and their mitochondrial redox states by imaging the Fp and NADH fluorescence signals of the premalignant group and the control group. From these images, we generated the redox indices: Fp, NADH, Fp redox ratio (Fp/(NADH + Fp)), Fp/NADH, and NADH/Fp and their standard deviations (SD) for further statistical analyses. We discovered that the redox states in the premalignant tissue (PTEN-null group) were highly heterogeneous as shown by the wide distribution in their histograms of the ratio indices (Figure 1). In contrast, the control group had relatively more homogenous redox states with smaller SDs of the Fp redox ratio, Fp/NADH, and NADH/Fp as evidenced by the narrower width in the corresponding histograms (Figure 2). In Figure 3, to show greater image details, we magnified the typical areas from the Fp redox ratio images in Figures 1 &2. It is clearly seen from Figure 3 that PTEN deficiency caused the tissue to be more heterogeneous in the Fp redox ratio with larger standard deviation.

To assess the tissue heterogeneity by the standard deviations of the redox indices, we used two statistical methods, Method I and Method II (see Materials and Methods section). Method I takes a global average across multiple imaging sections of a sample, whereas Method II includes results from each section for the analysis. Method I is expected to be less powerful in detecting statistical difference because of the loss of heterogeneity information with global averaging. The results are summarized in the lower panels of Tables 1 &2. Both methods revealed that the Fp redox ratio of the PTEN-null group were much more heterogeneous with significantly larger standard deviations compared to the control group. By Method I, the SD of the Fp redox ratio is 0.05 ± .02 and 0.10 ± .01 for the control and PTEN null group, respectively with p = 0.01 by the two-tailed t-test. By Method II, the SD of the Fp redox ratio is 0.06 ± .02 and 0.10 ± .01 for the control and PTEN-null group, respectively with p < 0.001, more significant than that of Method I.

**Table 1:** The redox indices (mean ± SD) and their standard deviations (mean ± SD) (Method I)

	**Fp**	**NADH**	**Fp Redox Ratio**	**Fp/NADH**	**NADH/Fp**
**Ctrl (N = 3, 9 sections)**	290 ± 88	568 ± 134	0.34 ± .02	0.53 ± .04	2.07 ± .12
**PTEN-null (N = 3, 14 sections)**	171 ± 27	294 ± 40	0.39 ± .07	0.73 ± .22	1.92 ± .60
**p**	0.13	0.06	0.34	0.25	0.71
	**SD**_**(Fp)**_	**SD**_**(NADH)**_	**SD**_**(Fp Redox Ratio)**_	**SD**_**(Fp/NADH)**_	**SD**_**(NADH/Fp)**_
**Ctrl (N = 3, 9 sections)**	78 ± 32	151 ± 37	0.05 ± .02	0.14 ± .06	0.44 ± .14
**PTEN-null (N = 3, 14 sections)**	82 ± 15	158 ± 64	0.10 ± .01	0.39 ± .14	0.82 ± .18
**p**	0.89	0.88	0.01	0.08	0.05

**Table 2:** The redox indices (mean ± SD) and their standard deviations (mean ± SD) (Method II)

	**Fp**	**NADH**	**Fp Redox Ratio**	**Fp/NADH**	**NADH/Fp**
**Ctrl (N = 3, 9 sections)**	298 ± 113	581 ± 181	0.31 ± .04	0.53 ± .09	2.06 ± .32
**PTEN-null (N = 3, 14 sections)**	167 ± 49	301 ± 101	0.37 ± .08	0.69 ± .26	2.01 ± .73
**p**	0.002	<0.001	0.01	0.003	0.17
	**SD**_**(Fp)**_	**SD**_**(NADH)**_	**SD**_**(Fp Redox Ratio)**_	**SD**_**(Fp/NADH)**_	**SD**_**(NADH/Fp)**_
**Ctrl (N = 3, 9 sections)**	82 ± 33	156 ± 45	0.06 ± .02	0.15 ± .06	0.47 ± .16
**PTEN-null (N = 3, 14 sections)**	81 ± 23	168 ± 72	0.10 ± .01	0.37 ± .15	0.85 ± .28
**p**	0.79	0.53	<0.001	<0.001	0.006

**Figure 4:**
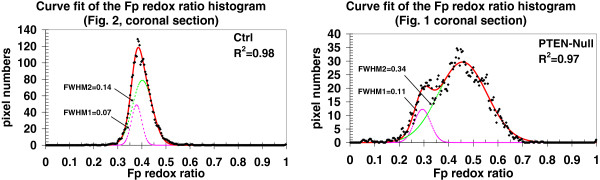
**Gaussian fit of the Fp redox ratio histogram.** Typical fits (red line) of the histograms of the Fp redox ratios (black dots) in the coronal sections in Figures 1 &2 by the sum of two Gaussian functions (green & pink lines). Left: control; Right: PTEN-null.

We also adopted a different approach to assessing the heterogeneity of the Fp redox ratio, where each histogram of the Fp redox ratios was fitted with two Gaussian functions (Figure 4) (see Materials and Methods section). Both fitting coefficients c_1_ and c_2_, thus the widths of the Gaussian curves FWHM_1_ and FWHM_2_ exhibited a significant difference between the two groups by both Method I and II (Table 3). FWHM or c depicts the bell shape of a Gaussian curve. A larger FWHM or c means the curve is “fatter”, otherwise “skinner”. Larger c_1_ and c_2_ in the Gaussian curves of Fp redox ratio delineates the wider distribution of the redox state or a larger standard deviation for the mean value of redox ratios. Therefore, the PTEN-null group having significantly larger c_i_ coefficients was much more heterogeneous in the redox state. There is no significance difference between two groups in either b_1_ or b_2_ representing the centers of the Fp redox ratio values of the two Gaussian peaks. The fitting coefficients a_1_ and a_2_ are proportional to the number of pixels or areas of each Gaussian fit and are tissue-size dependent. Their ratio, a_2_/a_1,_ which is the area ratio of the two Gaussian curves is a more meaningful comparison. There is no significant difference in a_2_/a_1_ between the two groups. Analysis performed with both Method I and II indicates no significant difference in a_2_/a_1_ between the two groups.

**Table 3:** **The fitting Gaussian coefficients (mean ± SD) of the Fp redox ratio**s

		**a**_**1**_	**a**_**2**_	**b**_**1**_	**b**_**2**_	**c**_**1**_	**c**_**2**_	**a**_**2**_**/a**_**1**_
**Method I**	**Ctrl (N = 3, 9 sections)**	54 ± 30	33 ± 24	0.31 ± .02	0.35 ± .02	0.032 ± .003	0.065 ± .01	0.72 ± .47
	**PTEN-null (N = 3, 14 sections)**	39 ± 15	24 ± 10	0.32 ± .06	0.37 ± .04	0.071 ± .006	0.12 ± .02	0.84 ± .22
	**p**	0.49	0.59	0.91	0.53	0.001	0.018	0.71
**Method II**	**Ctrl (N = 3, 9 sections)**	52 ± 36	36 ± 27	0.31 ± .03	0.34 ± .04	0.033 ± .01	0.07 ± .02	0.82 ± .50
	**PTEN-null (N = 3, 14 sections)**	38 ± 28	23 ± 16	0.32 ± .13	0.38 ± .12	0.074 ± .04	0.13 ± .04	0.84 ± .86
	**p**	0.067	0.04	0.39	0.13	0.004	0.001	0.87

### PTEN deletion shifted the pancreases to a more oxidized mitochondrial redox state

When taking tissue depth into account, the difference in Fp, NADH, the Fp redox ratio, and Fp/NADH between the two groups becomes statistically significant by Method II (Table 2). The two ratio indices, the Fp redox ratio and Fp/NADH became larger due to PTEN deletion, where the Fp redox ratio increased from 0.31 ± 0.04 to 0.37 ± .08 (p = 0.01) and Fp/NADH increased from 0.53 ± 0.09 to 0.69 ± 0.26 (p = 0.003). The increases indicate that the PTEN deletion had shifted the pancreases to an overall more oxidized redox state. In comparison, Method I does not show any significant difference in the redox ratio indices between the PTEN-null and control groups. In terms of concentration, the PTEN-null group has only about half amount of Fp (p = 0.002) or NADH (p < 0.001) compared with the control group (Method II).

## Discussion

### Possible link between the mitochondrial redox state alteration and cancer transformation

We imaged the mitochondrial redox indices of the PTEN-null mouse model using the redox scanner to look for potential new biomarkers for pre-malignancy detection. To support their rapid growth and survival in stressful and dynamic microenvironment, cancer cells constitutively activate their key oncogenic signaling pathways which converge to adapt/alter core cellular metabolism of all four major classes of macromolecules: carbohydrates, proteins, lipids, and nucleic acids. As one of the key oncogenic signaling pathways, PI3K/AKT pathway has profound effects on the metabolism of cancer cells [9]. It regulates glucose uptake and utilization and its activation renders cells dependent on high levels of glucose flux [15]. The downstream molecule NF-κB in the PI3K/AKT pathway controls the balance between the utilization of glycolysis and mitochondrial respiration [44]. Studies show that up to 60% of pancreatic cancers have either amplified or activated AKT2 kinase [45-47]. PTEN is a negative regulator of PI3K/AKT pathway. PTEN mutations amplify PI3K/AKT signaling and promote tumorigenesis. For example, it is shown that genomic deletion of PTEN is associated with prostate tumor metastatic potential [48]. However, the *in vivo* metabolic evaluation on the role of activated PI3-K/AKT signaling in the initiation or progression of pancreatic cancer has not been reported.

Our results showed that the PTEN-null pancreases had many regions with higher values of the Fp redox ratio (as typically seen in Figures 1 &3). The heterogeneity markers such as the SDs or the histogram widths (c_i_) of the Fp redox ratio, Fp/NADH, and NADH/Fp are significantly larger in the PTEN-null pancreases, whereas the tissues in the control group were relatively more homogeneous with significantly smaller SDs and smaller Gaussian curve width c_i_ (Tables 1, 2 and 3). Higher level of metabolic heterogeneity is consistent with previous observations on various pre-cancer tissues [37,38]. Therefore, it is likely that heterogeneous mitochondrial redox state is associated with cancer transformation. This indicates that (pre)malignant tissues may exhibit significant mitochondrial redox abnormality in localized spots before fully transformed.

We also observed lower levels of Fp and NADH in the PTEN-null group (Table 2). Decreased Fp and NADH is probably partially due to decreased cell density in premalignant tissues which had various sizes of ductal structures and cystic dilations [39]. The ratio indices that represent the metabolic and redox states of the tissues, Fp/NADH, NADH/Fp, or Fp/(Fp + NADH) and their standard deviations are less sensitive to the variability of cell density. A few other factors could affect Fp and NADH fluorescence signals, such as blood circulation and instrument fluctuations. Ratiometric indices such as the redox ratios are largely insensitive to these factors. Notably, only the SDs of the ratio indices distinguished between two groups whereas the SDs of Fp and NADH did not. This suggests that the heterogeneity of the ratio indices may be a better marker than the heterogeneity of Fp and NADH for the identification of premalignant tissues.

The result from this study also indicates that PTEN deletion shifted the tissue to a more oxidized state. Significantly higher Fp redox ratio and Fp/NADH were found in the PTEN-null group compared to the control group (Table 2). Higher Fp redox ratio or Fp/NADH was not reported previously by other researchers on various pre-cancer tissues [36-38]. For example, one of the studies imaged the redox ratios at various tissue depths for precancerous epithelia of oral cancer [37], and no significant difference was found in the redox ratios when comparing global averages between the precancerous and normal tissues. This is exactly what we have seen using Method I analysis. However, we saw statistically significant difference when using Method II that takes tissue depth as a covariate in GLM analysis. Another study [38] measured the 3D distribution of the redox state in fast-frozen human cervical tissues that typically consisted of an upper epithelium and a lower stroma. When compared to the normal tissue, the dysplastic epithelia had decreased redox ratio whereas the stroma appeared to have increased redox ratio (estimated based on their reported data [26]). However, it was not reported whether there was any difference in the overall averages of the redox ratios of the entire tissue block. The other study [36] measured the autofluorescence signals to investigate the redox state in the clinical cervical tissue samples that have been kept under temperatures above 0°C for 1.5-5 hours before imaging. Reduced redox ratios were found in the dysplastic epithelia of one third of the patient samples compared to the paired normal samples. During the long waiting period before imaging, the intrinsic fluorescence signal and the redox state in the tissue samples might have changed significantly and differential changes between the precancerous and the normal tissues might have occurred. In contrast, the mice’s pancreases used in our study were resected and dropped in liquid nitrogen within two seconds, and have been kept in liquid nitrogen for storage and during the redox scanning process. Their mitochondrial redox states should be the same as or very similar to the *in vivo* condition due to the snap-freezing procedure.

Nevertheless, the slightly higher Fp redox ratio in the PTEN-null group appears to be consistent with our previous studies on the redox states of the normal tissue and indolent and aggressive tumor tissues [26,32-34]. The normal muscle tissues had lower Fp redox ratio compared with the cancer tissues. The aggressive tumors exhibited a readily-recognized bi-modal distribution of their redox indices (e.g. Fp, NADH, Fp redox ratio) with substantial regions having significantly higher Fp redox ratio, i.e., more oxidized redox state, whereas the indolent tumors had relatively homogeneous and reduced redox states. The more aggressive the tumors were, the more heterogeneous they were and higher Fp redox ratio in the oxidized regions. Thus, the results of this study provide more evidence to a link between malignant transformation/progression and a more oxidized and heterogeneous mitochondrial redox state.

However, the role of the mitochondrial redox state in cancer transformation/progression remains largely unclear. Connections between the redox potentials (or NADH levels) and malignancy have been implicated in some studies [49-54]. High levels of free radicals/oxidants are known to generate oxidative damages and genetic mutations that may drive cancer transformation/progression. The mitochondrion is one of the major sources of free radical generation. Mitochondrial dysfunctions have been shown to induce overproduction of reactive oxygen species (ROS), which increases metastatic potential of cancer cells [51,55] and drives tumor transformation/progression in surrounding tissues in conjunction with oncogenic Ras [56]. However, ROS-independent pathways may also exist [57]. Nevertheless, high levels of free radicals/oxidants do not necessarily indicate oxidized redox potential. The high levels of oxidants in tumors are often counter-balanced by high levels of reductants such as vitamin C, reduced glutathione, and reduced nicotinamide adenine dinucleotide phosphate (NADPH) [53,58-60]. Cellular redox potential, i.e., the balance between reductants and oxidants, is mediated by multiple intracellular redox couples, e.g. NAD^+^/NADH, NADP^+^/NADPH, and oxidized/reduced glutathione. Redox biology is further complicated by the uneven distributions of redox systems in subcellular compartments (cytosolic, nuclear, mitochondrial, etc.). The mitochondrial redox potential may or may not be coupled with the cytosolic redox potential [61]. If we can accurately and precisely quantify the intracellular redox potential in each subcellular compartment, then it is possible to have a better understanding of the connections between mitochondrial redox state and cancer transformation/progression as evidenced by our studies.

### Importance of imaging intratumor heterogeneity by submillimeter imaging methods

It is known that concentrations of crucial nutrients such as glucose, glutamine, and oxygen are spatially and temporally heterogeneous in solid tumors [9,10]. Intratumor heterogeneity is an important factor for cancer diagnosis and treatment. The variability among subpopulations of tumor cells is well known for tumor progression and such heterogeneity is associated with malignancy and metastasis [62-65] as well as treatment failure [66]. Studies also showed that tissue heterogeneity is indicative of malignant phenotypes [67,68]. Imaging tumor heterogeneity may grade tumor invasive/metastatic potential and yield important prognostic and/or predictive biomarkers [32,33,69]. Promisingly, imaging pre-cancer tissues also revealed the existence of metabolic heterogeneity in dysplastic/premalignant tissues [36-38].

Since the tissue metabolic heterogeneity may be on a submillimeter scale [18], other metabolic biomarkers such as FDG-PET with a spatial resolution on the order of millimeters [70,71] are incapable of resolving it. Therefore, it is important to study tissues by using a high resolution imager. The redox scanner [24,27] we employed has a spatial resolution down to 50 × 50 × 20 μm^3^. As shown by the present study, the submillimeter high resolution redox imaging of tissue heterogeneity enhances the capability to identify biological factors that are important for cancer transformation but may not be seen by low resolution imaging techniques or the measurements of tissue homogenates. It should also be noted that the snap-freezing of the pancreases right after resection ensured the redox state of the frozen tissues is the same as or very similar to that of the *in vivo* condition.

### Selection of proper analysis methods to account for tissue heterogeneity

In our previous studies, when averaging over entire tumor, the heterogeneity information was largely lost, resulting in less significant or insignificant difference in Fp redox ratio between the aggressive and indolent tumors [32,33]. Consistently, we did not see significant difference in the Fp redox ratio between the premalignant and the control group after the global averaging for each pancreas using Method I that largely ignores the spatial heterogeneity of these redox indices. However, the redox images show that tissue heterogeneity exists in three dimensions in both the PTEN-null and control groups, with the PTEN-null group having a distinctly higher level of heterogeneity as evidenced by both image visualization (Figures 1, 2 and 3) and the quantifications of the standard deviations and widths of the Gaussian fits. The global averaging in Method I effectively removed the information on tissue heterogeneity and also reduced the number of independent data for statistical analysis. Thus, global average does not truly represent the heterogeneous nature of the diseased tissues, which may be critically important for distinguishing between groups.

Compared to Method I, Method II recognizes tissue heterogeneity in one of the three dimensions by using tissue depth (z-direction) as a covariate for the statistical analysis. Each tissue section has a tissue depth that is the distance between the top section and the specified section. Increased statistical significance was observed using Method II, yielding much smaller p values for the differences of the means between the PTEN-null and control groups. For example, a significant p value of 0.01 was reached for comparing the means of the Fp redox ratios from Method II whereas Method I failed to statistically differentiate Fp redox ratios between two groups. The difference between Method I and II shows the importance of selecting proper statistical analysis method for comparing multi-slice imaging results.

This result also shows the advantage of imaging measurements compared with the common practice that measures tissue homogenates where tissue has been ground and mixed to be homogenized [61,72,73]. We observed high heterogeneity of the redox state in both coronal and transversal planes of PTEN-null pancreases and along tissue depth. As heterogeneity is one of the very important characteristics of pathological tissues including precancerous tissues, the more tissue heterogeneity information is acquired, the better they are characterized. Suitable statistical analysis methods are also needed to account for the heterogeneity in three dimensions.

One of the shortcomings of the study is that the spatial resolution of the redox images of 100 μm used in this study is still not capable of fully resolving the pancreas premalignant lesions involving the highly proliferative ductal formations (size on the order of ~200 μm). In future, we may use CCD based redox imaging techniques to advance the study at a higher spatial resolution. We may also co-register the redox images with the histological staining and incorporate the in-depth studies on the biology to identify the sources of those more oxidized spots.

## Conclusions

In this paper we presented the redox imaging data with detailed analyses on the mitochondrial redox state of the normal pancreases and the PTEN-null pancreases that had developed premalignant lesions. The results showed that the PTEN deficiency had significantly altered the mitochondrial redox state of the pancreatic tissue. The mitochondrial redox state in pancreas became more oxidized and heterogeneous due to the deletion of the negative regulator of PI3K/AKT pathway. Such alteration in the mitochondrial redox state may provide possible biomarkers for early cancer detection. Although the current redox scanning method is applicable to clinical specimens, non- or minimal-invasive imaging methods for measuring tissue mitochondrial redox state *in vivo* should be developed in future.

Furthermore, this is the first study that showed possible link between pancreatic pre-malignancy and mitochondrial redox state alteration in pancreas. This study suggests that heterogeneous oxidization of the mitochondrial redox state in pancreatic tissue could be associated with cancer transformation. As the nature and importance of metabolic alterations in cancer can be masked by excess oxygen and nutrients in cultured cells, this study also shed light on cancer metabolism and the biology at tissue level.

## Materials and Methods

### Organ harvest and tissue sample preparation

Three pancreas-specific PTEN-null mice (Pdx-1-Cre;PTEN^lox/lox^) and three control mice (PTEN^lox/lox^) were prepared in Stanger’s laboratory [39]. The mice were approximately eight months old. The pancreases of the anesthetized mice were quickly resected during the open-chest surgery and dipped into liquid nitrogen within two seconds after removal. The samples for redox scanning were prepared as previously described [33,74,75]. Briefly, a snap-frozen pancreas was carefully placed into the mounting medium composed of ethanol-glycerol-water that was chilled below its freeze point -30°C and contained in a plastic screw closure of 24 mm diameter. Frozen reference standards (one NADH and one FAD in PBS buffer with known concentrations) were quickly mounted adjacent to the tissue. The embedded sample was then maintained in liquid nitrogen awaiting redox scanning. All samples were prepared the same way.

### Redox scanning

The redox scanning was performed using the redox scanner. Briefly, the sample immersed in liquid nitrogen was first milled flat with a surface area of ≥ 3 × 3 mm^2^. The bifurcated fiberoptic probe driven by a stepper motor scanned across the surface. The Fp channel excitation and emission filters were 430 ± 25 nm and 525 ± 32 nm, respectively; and those of NADH channel were 360 ± 13 nm and 430 ± 25 nm, respectively. Multiple sections at different milling depths (ranging from 200-2340 μm below the surface, i.e. the top section of a pancreas tissue) with spacing 100-400 μm were scanned for each pancreas (9 and 14 sections from the control and the PTEN-null group, respectively) with a scanning matrix being 128 × 128 and a step size of 100 μm. The reference standards were also scanned along with each tissue section.

### Data analysis

The acquired Fp and NADH signals were further analyzed with a customized MatLab® program to generate the needed images. The fluorescence intensities and concentrations of the reference standards were used to convert the tissue Fp and NADH fluorescence intensities to the corresponding nominal concentrations [33,74,75]. Pixels having SNR < 3.5 were excluded from the analysis. The mean values and the standard deviations (SD) of all redox indices, i.e. NADH, Fp, Fp redox ratio (Fp/(Fp + NADH)), Fp/NADH, and NADH/Fp were obtained for the region of interest (ROI) for each image section.

The statistical difference of these indices and their standard deviations between the PTEN-null and the control group was evaluated using two methods. Method I is to first average a redox index from a specific section to give the mean value of that section. The mean value for each section of the same pancreas was obtained in the same way. These mean values were then averaged to give the mean value of the redox index of that pancreas. Group mean value was obtained by averaging the mean value across three pancreases and the corresponding standard deviation was calculated. Two-taile, unpaired t-tests were performed to compare a specific redox index between the two groups, with p < 0.05 being considered statistically different. The same was performed for the standard deviation of each redox index. The results are summarized in Tables 1 &2. Although it is straightforward, this method loses information on the spatial heterogeneity of a specific redox index across different sections of a pancreas.

In addition to the heterogeneity in each tissue section, the mean value of each redox index also varies at different tissue depth. Tissue depth is the distance between the top section and the specified section. Method II uses the GLM Univariate analysis (IBM SPSS Statistics, ver. 20) with tissue depth as a covariate to evaluate whether the values of these redox indices depend on the PTEN status that is the independent variable. To perform this analysis, the mean value of each redox index from each tissue section was included as a sample of the dependent variables (e.g. NADH, Fp, or the Fp redox ratio). P < 0.05 is taken as statistically significant. The average tissue depth is 583 ± 257 μm and 927 ± 804 μm for the control and PTEN-null group, respectively. There is no statistical difference in the average of tissue depth between the two groups (p = 0.54). The results obtained by Method II are reported in Tables 1, 2 &3 as mean ± SD.

### Gaussian fit

The heterogeneity in Fp ratio was further quantitatively characterized using the curve fit approach in Matlab®. The sum of two Gaussian functions ( $f\left(x\right)=a_{1}e^{-\frac{{\left(x-b_{1}\right)}^{2}}{2{c_{1}}^{2}}}+a_{2}e^{-\frac{{\left(x-b_{2}\right)}^{2}}{2{c_{2}}^{2}}}$) was used to fit the Fp redox ratio histogram of each section, where parameter *a*_*i*_ is the height of the Gaussian function peak, *b*_*i*_ is the position of the centre of the peak, and *c*_*i*_ controls the width of the "bell" shape of the Gaussian curve (*i* = 1, 2). The full width at half maximum (FWHM) of a Gaussian function is given by $\mathrm{FWH}M_{i}=2\sqrt{2\ln2}c_{i}$. The results and their statistical analyses using the Method I and II are reported in Table 3.

## Competing interest

The authors declare no financial or non-financial interests to this study.

## Authors’ contributions

HNX carried out the redox scanning and performed the data analyses. SN assisted in organ harvest. HNX and LZL developed the data analysis methods. All authors participated in the design of the study. HNX drafted the manuscript. LZL and HNX revised the manuscript. SN reviewed the manuscript. HNX, SN, and LZL read and approved the final manuscript.

## Authors’ information

**This article is dedicated to the memory of late Dr. Britton Chance who conceived of the study.
